# Modulation of Circulating MicroRNAs Levels during the Switch from Clopidogrel to Ticagrelor

**DOI:** 10.1155/2016/3968206

**Published:** 2016-06-06

**Authors:** Annarita Carino, Salvatore De Rosa, Sabato Sorrentino, Alberto Polimeni, Jolanda Sabatino, Gianluca Caiazzo, Daniele Torella, Carmen Spaccarotella, Annalisa Mongiardo, Antonio Strangio, Carol Filippis, Ciro Indolfi

**Affiliations:** ^1^Division of Cardiology, Department of Medical and Surgical Sciences, Magna Græcia University, 88100 Catanzaro, Italy; ^2^URT-CNR, Department of Medicine, Consiglio Nazionale delle Ricerche, 88100 Catanzaro, Italy

## Abstract

*Background*. Circulating microRNAs are appealing biomarkers to monitor several processes underlying cardiovascular diseases. Platelets are a major source for circulating microRNAs. Interestingly, the levels of specific microRNAs were reported to correlate with the level of platelet activation. The aim of the present study was to test whether the treatment with the novel antiplatelet agent, ticagrelor, is associated with modulation in the levels of key platelet-derived microRNAs.* Methods and Results*. Patients were randomly selected from those participating in the SHIFT-OVER study, in which we had previously evaluated the effect of the therapeutic switch from clopidogrel to ticagrelor on platelet aggregation. Circulating levels of selected microRNAs were measured before and after the therapeutic switch from a dual antiplatelet therapy including acetylsalicylic acid (ASA) and clopidogrel to the more potent ticagrelor. Interestingly, the circulating levels of miR-126 (*p* = 0.030), miR-223 (*p* = 0.044), and miR-150 (*p* = 0.048) were significantly reduced, while the levels of miR-96 were increased (*p* = 0.038). No substantial differences were observed for the remaining microRNAs.* Conclusions*. Switching from a dual antiplatelet treatment with clopidogrel to ticagrelor is associated with significant modulation in the circulating levels of specific microRNAs. If confirmed in larger, independent cohorts, our results pave the way for the use of circulating microRNAs as biomarkers of platelets activity in response to specific pharmacological treatments.

## 1. Introduction

Despite the recent progress in the diagnosis and treatment, cardiovascular diseases are still the major source of morbidity and mortality worldwide. Furthermore, many pathophysiologic mechanisms still need to be disentangled, thus preventing the development of novel efficient and specific diagnostic and therapeutic strategies for a large number of patients. Platelets play an important role in the pathophysiology of cardiovascular diseases, especially in the development of their thrombotic complications, such as acute coronary syndromes (ACS) [1].

MicroRNAs (miRs) recently emerged as powerful regulators of biological processes, offering a further opportunity to better understand the biological mechanisms responsible for the development of cardiovascular diseases, including cellular function and cell-to-cell communication [2]. miRs are released into the bloodstream, offering the opportunity to monitor the biological status of the cardiovascular system through the measurement of the expression pattern of specific miRs in the blood [3, 4]. In particular, it became recently clear that platelets are a major source for circulating miRs [5]. Although muscle-enriched miRs (miR-499 and miR-133a) are released from the myocardium into the coronary circulation in ACS patients [6], miR-126, one of the most expressed platelet-related miRs [7], shows a negative concentration gradient across the coronary circulation, suggesting consumption by means of degradation, or tissue uptake or platelet adhesion/entrapment during the passage through the myocardium [6]. This latter finding is particularly interesting, given that levels of specific circulating miRs are associated with the degree of platelet activation [7] and can be therefore potentially used as biomarkers to monitor the efficacy of antiplatelet therapy.

On the basis of these findings, the aim of the present study was to evaluate whether the treatment with the novel P2Y12 antagonist, ticagrelor, is associated with modulation in the levels of key miRs associated with platelet function.

## 2. Materials and Methods

### 2.1. Study Population

Selected microRNAs were measured from plasma samples obtained from 16 patients from the SHIFT-OVER study [8], which enrolled 50 patients that were randomly assigned in a 1 : 1 fashion to either the “no load” group (switch to ticagrelor (90 mg BID) without loading dose) or the “load” group (switch to ticagrelor (180 mg) with loading dose). In particular, 8 patients were randomly selected from each of the following study groups: “no load” (group 1), including patients that were switched from clopidogrel to the maintenance dose of ticagrelor (90 mg* bis in die*) with no administration of the initial loading dose, and “load” (group 2), including patients that received a 180 mg loading dose, plus 90 mg* bis in die* (maintenance dose). To minimize the risk for selection bias, random selection of patients was performed using the online random number generator “random.org”. No statistical significant differences in clinically relevant characteristics were found between the randomly selected and nonselected patients. Patients from both groups received additional treatment with acetylsalicylic acid (ASA). Plasma samples were obtained at the following time points: (i) at baseline, when patients were on dual antiplatelet therapy including ASA 100 mg/die and clopidogrel sulphate 75 mg/die, and (ii) 24 hours after the patients had been shifted from clopidogrel to ticagrelor 90 mg* bis in die*. All patients provided written informed consent.

### 2.2. Blood Sampling

Blood samples were obtained through venous puncture. All blood samples were analyzed after a resting phase of 30 min. Plasma was obtained from blood samples harvested in 2.7 mL tubes containing 1.6 mg ethylenediaminetetraacetic acid/mL blood. We added 5 nmol/L* Caenorhabditis elegans* miR-39 (cel-miR-39) to the samples, during the extraction phase, to be used for normalization, as previously described [9]. Reverse transcription and quantitative (q)PCR were then performed with TaqMan microRNA assay kits, according to the instructions of the manufacturer. Values were normalized to cel-miR-39 and are expressed as 2^−(CT[microRNA]−CT[cel-miR-39])^. All measurements were run in duplicate and the mean CT values were calculated.

### 2.3. Platelet Aggregation Test

Whole blood platelet aggregometry was performed using a point-of-care Multiplate platelet analyzer, as previously described [8]. Briefly, 300 *μ*L of saline was mixed with whole blood at 37°C. After 3-minute incubation, 20 *μ*L of the agonist solution was added to obtain a final concentration of 6.4 *μ*mol/L adenosine diphosphate (ADP). Aggregometry results are reported as aggregation units (U), representing the mean values of 2 independent measurements. ΔU indicates the difference between aggregation levels measured at *T*_0_ (before the first administration of ticagrelor) and *T*_24_ (24 hours after the first ticagrelor administration).

### 2.4. Selection of Specific MicroRNAs

The specific microRNAs to be analyzed were selected among those that are known to be highly expressed in platelets or that had been previously associated with platelet activity. The following microRNAs were selected for the analysis: miR-233 and miR-126 are among the most highly expressed microRNAs in platelets and their levels are known to correlate with the level of platelet inhibition [5, 7, 10]; miR-150, miR-155, and miR-146a are key modulators for platelets production and activation [11, 12]; miR-96 is increased in subjects with hyporeactive platelets [13]; the levels of platelet miR-26b were found to be upregulated in patients with polycythemia [14].

### 2.5. Statistical Methods

Continuous variables are presented as the mean ± SD unless otherwise noted. Categorical variables were compared using the *χ* ^2^ test. Levels of circulating microRNAs between PRE and POST were compared using the Wilcoxon test (paired comparison) or the Mann-Whitney *U* test (unpaired comparisons). Statistical significance was assumed at *p* < 0.05. All statistical analyses were performed using SPSS software (version 20.0, Chicago, IL).

## 3. Results

Circulating microRNAs were measured in plasma samples obtained from 16 patients, selected from those included in the “no load” (*n* = 8) and the “load” (*n* = 8) groups from the previously published SHIFT-OVER study [8]. Baseline patients' characteristics are reported in Table 1. Levels of selected microRNAs measured in plasma samples obtained at baseline, when patients were still on dual antiplatelet therapy with ASA 100 mg/die and clopidogrel sulphate 75 mg/die (PRE), were compared to those measured 24 hours after the patients had been shifted from clopidogrel to ticagrelor 90 mg* bis in die* (POST).

**Figure 1:**
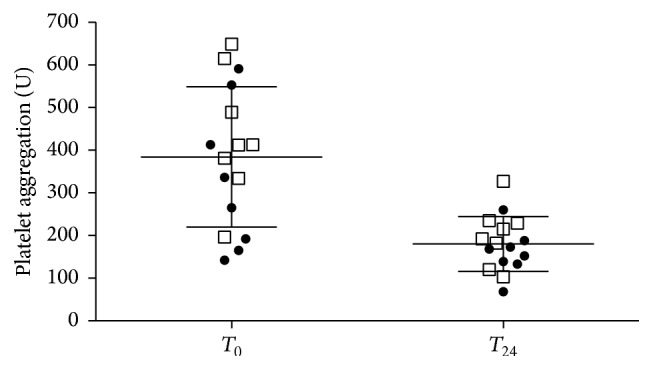
Results of platelet aggregation before (*T*_0_) and 24 h after (*T*_24_) the therapeutic switch from clopidogrel to ticagrelor. Rounded symbols indicate platelet aggregation levels in patients from the “no load” group, while squares represent patients from the “load” group.

Further significant inhibition of platelet aggregation was observed 24 h after the pharmacological switch from clopidogrel to ticagrelor (384 ± 154 U to 180 ± 64 U, *p* < 0.001). This effect was found both in the group of patients receiving the loading dose of ticagrelor (436 ± 148 U to 200 ± 70 U, *p* = 0.008) and in those receiving no loading dose during the switch from clopidogrel to ticagrelor (332 ± 173 U to 160 ± 54 U, *p* = 0.008). Results of platelet aggregation before and after the therapeutic switch are shown in Figure 1.

**Figure 2:**
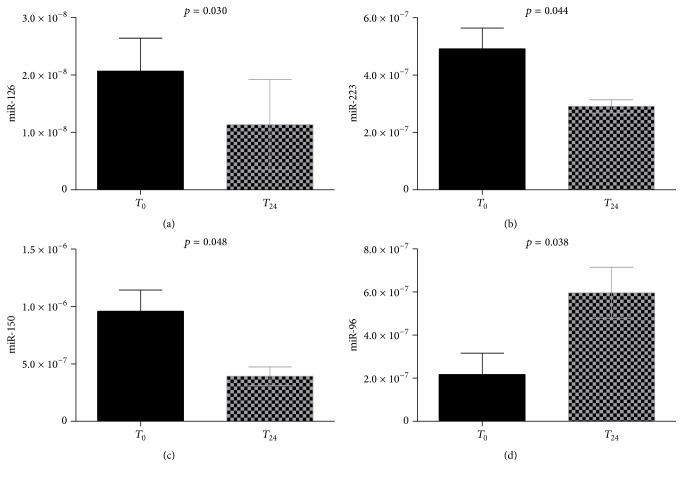
Levels of microRNAs. The bar graphs report mean and standard deviation values for miR-126 (a), miR-223 (b), miR-150 (c), and miR-96 (d) aggregation before (*T*_0_) and 24 h after (*T*_24_) the therapeutic switch from clopidogrel to ticagrelor.

Interestingly, comparing circulating levels of microRNAs measured after the therapeutic shift (POST) to those obtained at baseline (PRE), we found a 1.8-fold reduction for miR-126 (from 0.21 × 10^−7^ to 0.12 × 10^−7^, *p* = 0.030), a 2.1-fold reduction for miR-223 (from 0.49 × 10^−6^ to 0.29 × 10^−6^, *p* = 0.044), and a 2.8-fold reduction for miR-150 (from 0.96 × 10^−6^ to 0.39 × 10^−6^, *p* = 0.048). On the contrary, we observed a 2.6-fold increase (from 0.22 × 10^−6^ to 0.60 × 10^−6^) in the circulating levels of miR-96 (*p* = 0.038). Levels of these microRNAs at PRE and POST are displayed in Figure 2. On the other hand, no substantial differences in the circulating levels of miR-26b, miR-155, and miR-146a or the non-platelet-associated miR-190 were observed (all *p* = ns).

Of note, no significant differences were found in delta values (POST-PRE) between the two study groups (no load versus load) for miR-223 (*p* = 0.234), miR-150 (*p* = ns), miR-96 (*p* = 0.202), miR-155 (*p* = 0.250), or miR-26b (*p* = 0.400). On the contrary, a major decrease in miR-126 levels (3.6-fold versus 1.2-fold decrease; *p* = 0.002) was found in the group of patients randomized to receive a loading dose (load), as compared to group 1 (no load).

## 4. Discussion

The present study characterized the modulation in the circulating levels of specific microRNA, known to be associated with platelet function and/or activity. In fact, we found that plasma levels of some microRNAs were significantly decreased (miR-126, miR-223, and miR-150), while the levels of other molecules were increased (miR-96).

Very recently, a selective decrease in the plasma levels of specific microRNAs was reported in healthy volunteers and confirmed in patients on low-dose ASA treatment, after the administration of prasugrel [7]. Our results extend those observations to a hard clinical setting, with patients with acute coronary syndrome being already on dual antiplatelet therapy. In addition, while Willeit et al. evaluated the response to prasugrel [7], we demonstrated that a similar effect on circulating microRNAs can be observed with increasing intensity of platelet inhibition using ticagrelor (previous observation). This latter point makes our findings and previous ones more interesting, since they suggest that the same panel of microRNAs could be used to monitor the level of platelet inhibition, independently of the pharmacological agent administered to the patient.

Also interestingly, the same microRNAs that we found to be significantly decreased after treatment with ticagrelor in the present study had been associated with the risk of acute myocardial infarction in previous studies [15].

In addition to the potential use as biomarkers, the observed modulation in the levels of specific microRNAs in response to increasing platelet inhibition in patients with acute coronary syndrome represents a further confirmation of the pathophysiological role played by platelet-derived microRNAs in cardiovascular diseases. For example, we previously observed a negative concentration gradient across the coronary circulation of miR-126, suggesting platelet adhesion/entrapment in the myocardium. These results, together with the finding of high levels of miR-126 in human platelets, led to the hypothesis that it could play a role in the development of cardiovascular disease. According to this hypothesis, it was recently demonstrated that platelet-derived microparticles are able to deliver quanta of miR-126 to macrophages, with consequent shift of their functional profile towards a phagocytic phenotype [16]. Consequently, the reduction in the circulating levels of platelet-derived miR-126 could have a major impact on cardiovascular diseases. In this regard, our finding of a more pronounced reduction of miR-126 levels after the therapeutic switch to ticagrelor in the group of patients randomized to receive a loading dose (load) is particularly intriguing, since it suggests that measurement of microRNAs could provide additional information over platelet aggregation tests.

It was recently reported that platelet hyperreactivity is associated with an increased expression of VAMP8/endobrevin, a v-SNARE with a key role in platelet degranulation. Interestingly, VAMP8 is a known target of miR-96 [13]. Hence, the increase in miR-96 levels that we observed in POST samples, after the switch to the newer and more efficient P2Y12 inhibitor, is quite interesting and, together with previous evidence that miR-96 targets VAMP8, suggests the involvement of this specific microRNA in the modulation of platelet activation.

The complex but interesting relationship between the levels of platelet-derived miR-223 and the degree of platelet inhibition is well worth a comment. In fact, since miR-223 targets the gene for the ADP receptor P2Y12, it plays a key role in platelet activation and responsiveness to therapy. In fact, miR-223 levels were associated with high on-treatment platelet reactivity [10, 17]. However, for the same reasons, miR-223 is seemingly not an optimal marker for platelet reactivity, since its levels not only depend on the degree of stimulation/inhibition of the P2Y12 ADP receptor, but also modulate its expression levels.

Though very appealing, some issues still limit the introduction of circulating microRNAs into clinical practice. Potential limitations of their use include the intrinsic complexity associated with the use of qPCR for the measurement of microRNA levels in plasma. In fact, the relatively low expression level for some microRNAs, the absence of a well-validated “housekeeping” microRNA for normalization, and the elevated costs and long analytical times currently hamper the use of circulating microRNAs as biomarkers in clinical practice. However, several alterative detection methods are being developed and tested with varying results. Among the others, the use of nanosensor in association with microfluidic filters could allow the reliable and fast label-free detection of specific microRNAs with substantially lower costs compared to currently used methods [18, 19]. It is known that platelet microRNAs have good intraindividual stability, with larger interindividual variability [20]. The results reported in the present study are based on paired analyses of two different measurements obtained from the plasma of the same patient at two different time points. Consequently, the effect of the switch to a more potent antiplatelet agent on circulating microRNAs in the present study was intrinsically normalized for individual baseline levels. In this respect, it is not predictable whether a single measurement could be actually helpful in individual patients. Furthermore, despite random selection of patients from the SHIFT-OVER population and application paired analysis that reduces the influence of moderator variables, the risk for selection bias cannot be excluded, given the limited sample size. Though intriguing, findings of the present study do not represent conclusive evidence on the most appropriate drug posology in the clinical setting. Future studies should be specifically designed to evaluate this aspect.

In conclusion, switching from a dual antiplatelet treatment with clopidogrel to ticagrelor is associated with significant modulation in the levels of specific platelet-associated circulating microRNAs. If confirmed in larger, independent cohorts, our results pave the way for the use of circulating microRNAs as biomarkers of platelets activity in response to specific pharmacological treatments.

## Figures and Tables

**Table 1:** Baseline patients' characteristics.

Patients' characteristics	No load (*n* = 8)	Load (*n* = 8)
Age (mean ± SD)	61 ± 12	59 ± 7
Males/females	6/2	7/1
Family history of CVD	12.5%	12.5%
Acute coronary syndrome		
UA/NSTEMI (%)	37.5%	62.5%
STEMI (%)	62.5%	37.5%
Hypertension (%)	37.5%	37.5%
Multivessel disease	87.5%	100%
Diabetes mellitus (%)	25%	50%
Smokers (%)	62.5%	25%
Previous AMI (%)	12.5%	25%
Chronic kidney disease (%)	0%	0%

UA: unstable angina; NSTEMI: non-ST segment elevation acute myocardial infarction; STEMI: ST segment elevation acute myocardial infarction; AMI: acute myocardial infarction.
